# Dynamics of starvation and recovery predict extinction risk and both Damuth’s law and Cope’s rule

**DOI:** 10.1038/s41467-018-02822-y

**Published:** 2018-02-13

**Authors:** Justin D. Yeakel, Christopher P. Kempes, Sidney Redner

**Affiliations:** 1 School of Natural Sciences, University of California, Merced, CA 95340 USA; 2 The Santa Fe Institute, 1399 Hyde Park Road, Santa Fe, NM 87501 USA

## Abstract

The eco-evolutionary dynamics of species are fundamentally linked to the energetic constraints of their constituent individuals. Of particular importance is the interplay between reproduction and the dynamics of starvation and recovery. To elucidate this interplay, here we introduce a nutritional state-structured model that incorporates two classes of consumers: nutritionally replete, reproducing consumers, and undernourished, nonreproducing consumers. We obtain strong constraints on starvation and recovery rates by deriving allometric scaling relationships and find that population dynamics are typically driven to a steady state. Moreover, these rates fall within a “refuge” in parameter space, where the probability of population extinction is minimized. We also show that our model provides a natural framework to predict steady state population abundances known as Damuth's law, and maximum mammalian body size. By determining the relative stability of an otherwise homogeneous population to a competing population with altered percent body fat, this framework provides a principled mechanism for a selective driver of Cope’s rule.

## Abstract (web summary)

Energetic constraints produce a fundamental tradeoff in starvation and recovery rates, impacting eco-evolutionary dynamics. Here, Yeakel et al. develop a nutritional state-structured model that predicts population size as a function of body mass known as Damuth’s law, and a mechanism for Cope’s rule, the evolutionary trend towards larger body mass.

## Introduction

The behavioral ecology of all organisms is influenced by their energetic states, which directly impacts how they invest reserves in uncertain environments. Such behaviors are generally manifested as tradeoffs between investing in somatic maintenance or allocating energy toward reproduction^1–3^. The timing of these behaviors responds to selective pressure, as the choice of the investment impacts future fitness^4,5^. The influence of resource limitation on an organism’s ability to maintain its nutritional stores may lead to repeated delays or shifts in reproduction over the course of an organism’s life.

The balance between (a) somatic growth and maintenance, and (b) reproduction depends on resource availability^6^. For example, reindeer invest less in calves born after harsh winters (when the mother’s energetic state is depleted) than in calves born after moderate winters^7^. Many bird species invest differently in broods during periods of resource scarcity^8,9^, sometimes delaying or even foregoing reproduction for a breeding season^1,10,11^. Even freshwater and marine zooplankton have been observed to avoid reproduction under nutritional stress^12^, and those that do reproduce have lower survival rates^2^. Organisms may also separate maintenance and growth from reproduction over space and time: many salmonids, birds, and some mammals return to migratory breeding grounds to reproduce after one or multiple seasons in resource-rich environments where they accumulate reserves^13–15^.

Physiology also plays an important role in regulating reproductive expenditures during periods of resource limitation. Many mammals (47 species in ten families) exhibit delayed implantation, whereby females postpone fetal development until nutritional reserves can be accumulated^16,17^. Many other species (including humans) suffer irregular menstrual cycling and higher abortion rates during periods of nutritional stress^18,19^. In the extreme case of unicellular organisms, nutrition directly controls growth to a reproductive state^3,20^. The existence of so many independently evolved mechanisms across such a diverse suite of organisms highlights the near-universality of the fundamental trade-off between somatic and reproductive investment.

Including individual energetic dynamics^21^ in a population-level framework^21,22^ is challenging^23^. A common simplifying approach is the classic Lotka–Volterra (LV) model, which assumes that consumer population growth rate depends linearly on resource density^24^. Here, we introduce an alternative approach—the nutritional state-structured model (NSM)—that accounts for resource limitation via explicit starvation. In contrast to the LV model, the NSM incorporates two consumer states: hungry and full, with only the former susceptible to mortality and only the latter possessing sufficient energetic reserves to reproduce. Additionally, we incorporate allometrically derived constraints on the timescales for reproduction^3^, starvation, and recovery. Our model makes several basic predictions: (i) the dynamics are typically driven to a refuge far from cyclic behavior and extinction risk, (ii) the steady-state conditions of the NSM accurately predict the measured biomass densities for mammals described by Damuth’s law^25–28^, (iii) there is an allometrically constrained upper bound for mammalian body size, and (iv) the NSM provides a selective mechanism for the evolution of larger body size, known as Cope’s rule^29–32^.

## Results

### Nutritional state-structured model

We begin by defining the nutritional state-structured population model, where the consumer population is partitioned into two states: (a) an energetically replete (full) state *F*, where the consumer reproduces at a constant rate *λ* and does not die from starvation, and (b) an energetically deficient (hungry) state *H*, where the consumer does not reproduce but dies by starvation at rate *μ*. The dynamics of the underlying resource *R* are governed by logistic growth with an intrinsic growth rate *α* and a carrying capacity *C*. The rate at which consumers transition between states and consume resources is dependent on their number, the abundance of resources, the efficiency of converting resources into metabolism, and how that metabolism is partitioned between maintenance and growth purposes. We provide a physiologically and energetically mechanistic model for each of these dynamics and constants (Methods), and show that the system produces a simple nondimensional form that we describe below.

Consumers transition from the full state *F* to the hungry state *H* at a rate *σ*—the starvation rate—and also in proportion to the absence of resources (1−*R*) (the maximum resource density has been nondimensionalized to 1; see Methods). Conversely, consumers recover from state *H* to state *F* at rate *ξρ* and in proportion to *R*, where *ξ* represents a ratio between maximal resource consumption and the carrying capacity of the resource. The resources that are eaten by hungry consumers (at rate *ρR*+*δ*) account for their somatic growth (*ρR*) and maintenance (*δ*). Full consumers eat resources at a constant rate *β* that accounts for maximal maintenance and somatic growth (see Methods for mechanistic derivations of these rates from resource energetics). The NSM represents an ecologically motivated fundamental extension of the idealized starving random walk model of foraging, which focuses on resource depletion, to include reproduction and resource replenishment^33–35^, and is a more general formulation than previous models that incorporate starvation^36,37^.

In the mean-field approximation, in which the consumers and resources are perfectly mixed, their densities are governed by the rate equations

1 $$\begin{array}{*{20}{l}} {\dot F} \hfill & { = \lambda F + \xi \rho RH - \sigma \left( {1 - R} \right)F,} \hfill \\ {\dot H} \hfill & { = \sigma \left( {1 - R} \right)F - \xi \rho RH - \mu H,} \hfill \\ {\dot R} \hfill & { = \alpha \left( {1 - R} \right)R - \left( {\rho R + \delta } \right)H - \beta F.} \hfill \end{array}$$

This system of nondimensional equations follows from a set of first-principle relationships for resource consumption and growth (see Methods for a full derivation and the dimensional form). Notice that the total consumer density *F*+*H* follows $\dot F + \dot H = \lambda F - \mu H$. This resembles the equation of motion for the predator density in the LV model^38^, except that the resource density does not appear in the growth term. The rate of reproduction is independent of resource density because the full consumer partitions a constant amount of energy toward reproduction, whereas a hungry consumer partitions no energy toward reproduction. Similarly, the consumer maintenance terms *(δH* and *βF*) are also independent of resource density because they represent a minimal energetic requirement for consumers in the *H* and *F* state, respectively.

### Steady states of the NSM

From the single internal fixed point (Eq. 5, see Methods), an obvious constraint on the NSM is that the reproduction rate *λ* must be less than the starvation rate *σ*, so that the consumer and resource densities are positive. The condition *σ* *=* *λ* represents a transcritical (TC) bifurcation^39^ that demarcates a physical from an unphysical (negative steady-state densities) regime. The biological implication of the constraint *λ* < *σ* has a simple interpretation—the rate at which a macroscopic organism loses mass due to lack of resources is generally much faster than the rate of reproduction. As we will discuss below, this inequality is also a natural consequence of allometric constraints for organisms within empirically observed body size ranges.

**Fig. 1:**
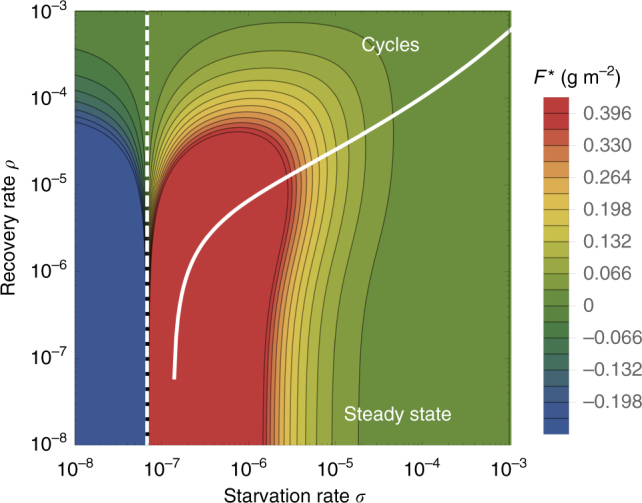
The transcritical (TC; dashed line) and Hopf bifurcation (solid line) as a function of the starvation rate *σ* and recovery rate *ρ* for a 100-g consumer. These bifurcation conditions separate parameter space into unphysical (left of the TC), cyclic, and steady-state dynamic regimes. The colors show the steady-state densities for the energetically replete consumer F^*^

In the physical regime of *λ < σ*, the fixed point (Eq. 5) may either be a stable node or a limit cycle (Fig. 1). In continuous-time systems, a limit cycle arises when a pair of complex conjugate eigenvalues crosses the imaginary axis to attain positive real parts^40^. This Hopf bifurcation is defined by Det(**S**) = 0, where **S** is the Sylvester matrix, which is composed of the coefficients of the characteristic polynomial of the Jacobian matrix^41^. As the system parameters are tuned to be within the stable regime, but close to the Hopf bifurcation, the amplitude of the transient cycles becomes large. Given that ecological systems are constantly being perturbed^42^, the onset of transient cycles, even though they decay with time in the mean-field description, can increase extinction risk^43–45^.

When the starvation rate *σ* >> *λ*, a substantial fraction of the consumers is driven to the hungry nonreproducing state. Because reproduction is inhibited, there is a low steady-state consumer density and a high steady-state resource density. However, if *σ*/*λ*→1 from above, the population is overloaded with energetically replete (reproducing) individuals, thereby promoting transient oscillations between the consumer and resource densities (Fig. 1). If the starvation rate is low enough that the Hopf bifurcation is crossed, these oscillations become stable. This threshold occurs at higher values of the starvation rate as the recovery rate *ρ* increases, such that the range of parameter space giving rise to cyclic dynamics also increases with higher recovery rates.

### The allometry of extinction risk

While there are no a priori constraints on the parameters in the NSM, we expect that each species should be restricted to a distinct portion of the parameter space. We use allometric scaling relations to constrain the covariation of rates in a principled and biologically meaningful manner (Methods). Allometric scaling relations highlight common constraints and average trends across large ranges in body size and species diversity. Many of these relations can be derived from a small set of assumptions. In Methods, we describe our framework to determine the covariation of timescales and rates across a range of body sizes for each of the key parameters of our model (cf. ref. ^46^).

**Fig. 2:**
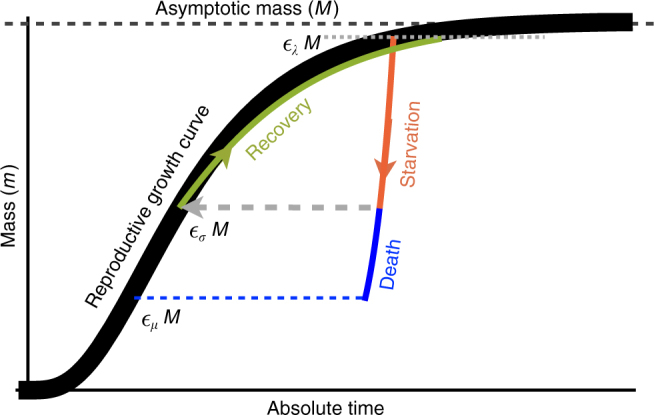
The growth trajectory over absolute time of an individual organism as a function of body mass. Initial growth follows the black trajectory to an energetically replete reproductive adult mass of *m* = *ε*_*λ*_*M* (Methods). Starvation follows the red trajectory to *m* = *ε*_*σ*_*ε*_*λ*_*M*. Recovery follows the green curve to the replete adult mass, where this trajectory differs from the original growth because only fat is being regrown that requires a longer time to reach *ε*_*λ*_*M*. Alternatively, death from starvation follows the blue trajectory to *m* = *ε*_*μ*_*ε*_*λ*_*M*

Nearly all of the rates described in the NSM are determined by consumer metabolism, which can be used to describe a variety of organismal features^47^. We derive, from first principles, the relationships for the rates of reproduction, starvation, recovery, and mortality as a function of an organism’s body size and metabolic rate (Methods). Because we aim to explore the starvation-recovery dynamics as a function of an organism’s body mass *M*, we parameterize these rates in terms of the percent gain and loss of the asymptotic (maximum) body mass, *εM*, where different values of *ε* define different states of the consumer (Fig. 2; see Methods for derivations of allometrically constrained rate equations). Although the rate equations (1) are general and can in principle be used to explore the starvation-recovery dynamics for most organisms, here, we focus on allometric relationships for terrestrial-bound lower-trophic-level endotherms (Table 1), specifically herbivorous mammals, which range from a minimum of *M* ≈ 1 g (the Etruscan shrew *Suncus etruscus*) to a maximum of *M* ≈ 10^7^ g (the early Oligocene Indricotheriinae and the Miocene Deinotheriinae). Investigating other classes of organisms would simply involve altering the metabolic exponents and scalings associated with *ε*. Moreover, we emphasize that our allometric equations describe mean relationships and do not account for the (sometimes considerable) variance associated with individual species. We note that including additional allometrically scaled mortality terms to both *F* and *H* does not change the form of our model nor impact our quantitative findings (Supplementary Note 1).

**Table 1:** Parameter values for mammals

Definition	Parameter	Value	References
Asymptotic adult mass	*M*	(g)	
Initial mass of an organism	*m*_0_	(g)	
Metabolic rate scaling exponent	*η*	3/4	^64–66^
Metabolic normalization constant	*B*_0_	0.047 (W g^−0.75^)	^66^
Initial mass scaling exponent	*υ*	0.92	^74,75^
Initial mass scaling normalization constant	*n*_0_	0.097 (g^1−*υ*^)	^74,75^
Fat mass scaling exponent	*γ*	1.19	^78^
Fat scaling normalization constant	*f*_0_	0.02 (g^1−*η*^)	^78^
Muscle mass scaling exponent	*ζ*	1.00	^79^
Muscle scaling normalization constant	*u*_0_	0.38 (g^1−*ζ*^)	^79^
Energy to synthesize a unit of mass	*E*_m_	5774 (J g^−1^)	^64–66^
Energy to synthesize a unit of mass during recovery	*E*′_m_	7000 (J g^−1^)	^66,76^
Specific resource growth rate	*α*	9.45×10^−9^ (s^−1^)	See text
Fraction of asymptotic mass representing full state	*ε*_*λ*_	0.95	^64^
Fraction of asymptotic mass representing starving state	*ε*_*σ*_	1−*f*_0_*M*^*γ*−1^	See text
Fraction of asymptotic mass representing death	*ε*_*μ*_	$1 - \frac{{f_{\mathrm{0}}M^\gamma + u_{\mathrm{0}}M^\zeta }}{M}$	See text
Carrying capacity (maximum density) of resources	*C*	(g m^−2^)	
Half-saturation constant	*k*	(g m^−2^)	
Normalized carrying capacity	*ξ*	*C*/*k *≈ 2	
Reproductive fecundity	*ν*	2	

As the allometric derivations of the NSM rate laws reveal (Methods), starvation and recovery rates are not independent parameters, and the biologically relevant portion of the phase space shown in Fig. 1 is constrained via covarying parameters. Given the parameters of terrestrial endotherms, we find that the starvation rate *σ* and the recovery rate *ρ* are constrained to lie within a small region of potential values for the known range of body size *M*. Indeed, starvation and recovery rates across all values of *M* fall squarely in the steady-state region at some distance from the Hopf bifurcation. This suggests that cyclic population dynamics should be rare, particularly in resource-limited environments.

**Fig. 3:**
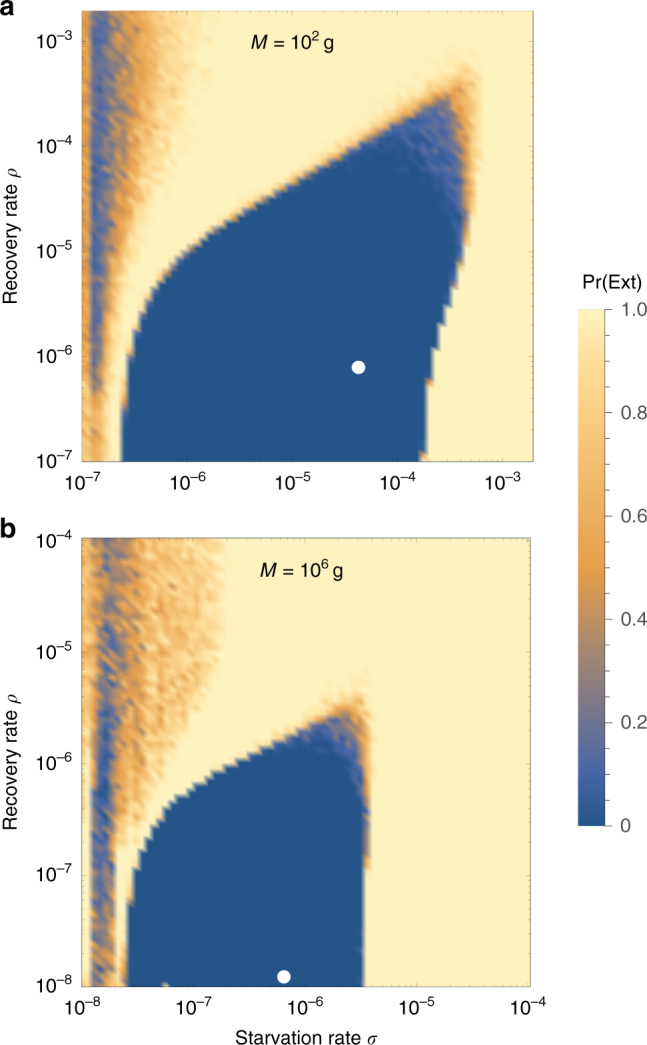
Probability of extinction for a consumer with **a** *M* = 10^2^ g and **b** *M* = 10^6^ g as a function of the starvation rate *σ* and recovery rate *ρ*, where the initial density is given as (XF^*^, XH^*^, R^*^), where *X* is a random uniform variable in [0, 2]. Note the change in scale in **b**. Extinction is defined as the population trajectory falling below 0.2× the allometrically constrained steady state. The white points denote the allometrically constrained starvation and recovery rates for consumers of each body size

Higher rates of starvation result in a larger flux of the population to the hungry state. In this state, reproduction is absent, thus increasing the likelihood of extinction. From the perspective of population survival, it is the rate of starvation relative to the rate of recovery that determines the long-term dynamics of the various species (Fig. 1). We therefore examine the competing effects of cyclic dynamics vs. changes in steady-state density on extinction risk, both as functions of *σ* and *ρ*. To this end, we computed the probability of extinction, where we define extinction as a population trajectory falling below one-fifth of the allometrically constrained steady state at any time between *t* = 10^8^ and *t* = 10^10^. This procedure was repeated for 50 replicates of the continuous-time system shown in Eq. 1 for organisms with mass ranging from 10^2^ to 10^6^ g. In each replicate, the initial densities were chosen to be (XF^*^, XH^*^, R^*^), with *X* a random variable uniformly distributed in [0, 2]. By allowing the rate of starvation to vary, we assessed extinction risk across a range of values for *σ* and *ρ* between ca. 10^−8^ and 10^−3^. Higher rates of extinction correspond to both large *σ* if *ρ* is small, and large *ρ* if *σ* is small. In the former case, increased extinction risk arises because of the decrease in the steady-state consumer population density (Figs. 1, 3). In the latter case, the increased extinction risk results from higher-amplitude transient cycles as the system nears the Hopf bifurcation (Fig. 3). This interplay creates an “extinction refuge”, such that for a constrained range of *σ* and *ρ*, extinction probabilities are minimized.

We find that the allometrically constrained values of *σ* and *ρ*, each representing different trajectories along the ontogenetic curve (Fig. 2), fall squarely within the extinction refuge across a range of *Μ* (Fig. 3a, b, white points). These values are close enough to the Hopf bifurcation to avoid low steady-state densities, yet distant enough to avoid large-amplitude transient cycles. Allometric values of *σ* and *ρ* fall within this relatively small window, which supports the possibility that a selective mechanism has constrained the physiological conditions driving starvation and recovery rates within populations. Such a mechanism would select for organism physiology that generates appropriate *σ* and *ρ* values that minimize extinction risk. This selection could occur via the tuning of body fat percentages, metabolic rates, and/or biomass maintenance efficiencies. We also find that as body size increases, the size of the low extinction-risk parameter space shrinks (Fig. 3b), suggesting that the population dynamics for larger organisms are more sensitive to variability in physiological rates. This finding is in accordance with, and may serve as contributing support for, observations of increased extinction risk among larger mammals^48^.

### Damuth’s law and body size limits

**Fig. 4:**
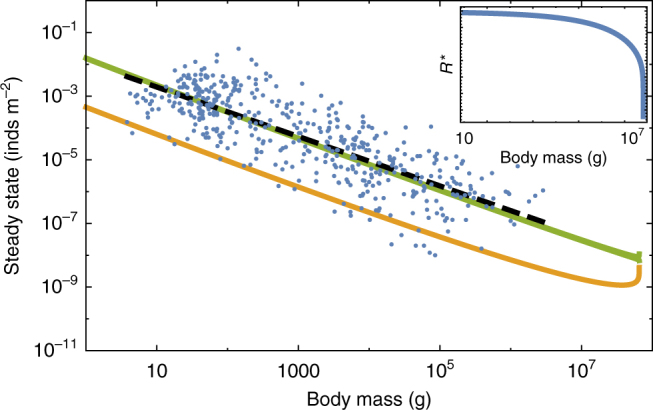
Consumer steady states F^*^ (green) and H^*^ (orange) as a function of body mass along with the data from Damuth^25^. Inset: resource steady state R^*^ as a function of consumer body mass

The NSM correctly predicts that smaller species have larger steady-state population densities (Fig. 4). Similar predictions have been made for carnivore populations using alternative consumer-resource models^49^. Moreover, we show that the NSM provides independent theoretical support for Damuth’s law^25–28^. Damuth’s law shows that species abundances, Ν^*^, follow Ν^*^ = 0.01*Μ*^−0.78^ (g m^−2^). Figure 4 shows that both F^*^ and Η^*^ scale as *M*^−*η*^, with *η* ≈ 3/4, over a wide range of organismal sizes and that F^*^+ Η^*^ closely matches the best fit to Damuth’s data. Remarkably, this result illustrates that the steady-state values of the NSM combined with the derived timescales naturally give rise to Damuth’s law. While the initial metabolic studies supporting Damuth’s law provide arguments for the value of the exponent^25,26^ (Supplementary Note 2), our model predicts not only the exponent but also the normalization constant dependencies by explicitly including the resource dynamics and the parameters that determine growth and consumption. These predictions are complementary to recent work that also predicts the exponent and normalization constant of density relationships from the detailed allometries of reproduction, capture area, conversion efficiency, and mortality within predator–prey dynamic models^49,50^. It should be noted that density relationships of individual clades follow a more shallow scaling relationship than that predicted by Damuth’s law^28^. In the context of our model, this finding suggests that future work may be able to anticipate these shifts by accounting for differences in the physiological parameters associated with each clade.

With respect to predicted steady-state densities, the population sizes of both* F* and *H* go to zero at a finite body size, where the steady-state resources also vanish (Fig. 4). This behavior is governed by the body size at which *f*_0_*M*^γ−1^+*u*_0_*M*^*ζ*−1^ = 1 (Supplementary Note 3) that causes the death rate to vanish, *μ* = 0, and corresponds to (F^*^, H^*^, R^*^) = (0, 0, 0). Just before this point H^*^ becomes large, in  accordance with the asymptotic behavior in Fig. 4. This point predicts an upper bound on mammalian body size at *M*_max_ = 6.54 × 10^7^ g. Moreover, *M*_max_, which is entirely determined by the population-level consequences of energetic constraints, is within an order of magnitude of the maximum body size observed in the North American mammalian fossil record^29^, as well as the mass predicted from an evolutionary model of body size evolution^30^. We emphasize that the asymptotic behavior and predicted upper bound depend only on the scaling of body composition and are independent of the resource parameters. The prediction of an asymptotic limit on mammalian size parallels work on microbial life where an upper and lower bound on bacterial size, and an upper bound on single-cell eukaryotic size, is predicted from similar growth and energetic scaling relationships^3,51^. It has also been shown that models that incorporate the allometry of hunting and resting combined with foraging time predicts a maximum carnivore size between 7 × 10^5^ and 1.1 × 10^6^ g^52,53^. Similarly, the maximum body size within a particular lineage has been shown to scale with the metabolic normalization constant^54^. This complementary approach is based on the balance between growth and mortality, and suggests that future connections between the scaling of fat and muscle mass should systematically be connected with *B*_0_ when comparing lineages.

### A mechanism for Cope’s rule

Metabolite transport constraints are widely thought to place limits on biological scaling^47,55^ and thereby lead to specific predictions on the minimum possible body size for organisms^56^. Above this bound, a number of energetic and evolutionary mechanisms have been explored to assess the costs and benefits associated with larger body masses, particularly for mammals. One important such example is the “fasting endurance hypothesis”, which contends that larger body size, with consequent lower metabolic rates and increased ability to maintain more endogenous energetic reserves, may buffer organisms against environmental fluctuations in resource availability^57^. Over evolutionary time, terrestrial mammalian lineages show a significant trend toward larger body size—Cope’s rule^29–32^. It is thought that within-lineage drivers generate selection toward an optimal upper bound of roughly 10^7^ g^29^, a value that is likely limited by higher extinction risk for large taxa over longer timescales^30^. These trends are thought to be driven by a combination of climate change and niche availability^32^; however, the underpinning energetic costs and benefits of larger body sizes, and how they influence dynamics over ecological timescales, have not been explored.

The NSM predicts that the steady-state resource density R^*^ decreases with increasing body size of the consumer population (Fig. 4, inset), and classic resource competition theory predicts that the species surviving on the lowest resource abundance will outcompete others^58–60^. Thus, the combined NSM steady-state dynamics and allometric timescales (Eq. 7) predict that larger mammals have an intrinsic competitive advantage given a common resource.

However, the above resource relationships do not offer a mechanism for how body size is selected. We directly assess competitive outcome between two closely related species: a resident species of mass *M*, and a competing species (denoted by ′) where individuals have a different proportion of body fat such that *M*′ = *M*(1+*χ*). For *χ* < 0, the competing individuals have fewer metabolic reserves than the resident species and vice versa for *χ* > 0. For the allowable values of *χ* (Methods), the mass of the competitor *M′* should exceed the minimal amount of body fat, 1 + *χ* > *ε*_*σ*_, and the adjusted time to reproduce must be positive, which, given Eq. 7, implies that $1 - \varepsilon _{\mathrm{\lambda }}^{1 - \eta }\left( {1 + \chi } \right)^{1 - \eta } \, > \, 0$. These conditions imply that $\chi \in ( - f_0M^{\gamma - 1},1/\varepsilon _{\mathrm{\lambda }} - 1)$ where the upper bound approximately equals 0.05 and the lower bound is mass-dependent. The modified mass of the competitor leads to altered rates of starvation *σ*(*M*′), recovery *ρ*(*M*′), and the maintenance of both starving *δ*(*M*′) and full consumers *β*(*M*′) (see Methods for derivations of competitor rates). Importantly, *ε*_*σ*_, which determines the point along the growth curve that defines the body composition of starved foragers, is assumed to remain unchanged for the competing population.

**Fig. 5:**
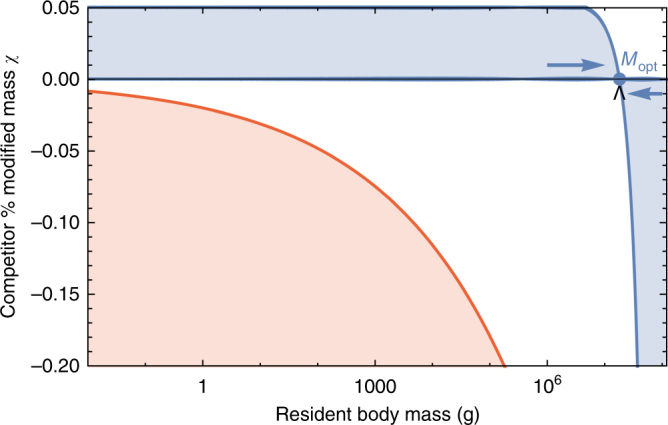
Competitive outcomes for a resident species with body mass *M* vs. a closely related competing species with modified body mass *M*′ = *M*(1 + *χ*). The blue region denotes proportions of modified mass *χ* resulting in exclusion of the resident species. The red region denotes values of *χ* that result in a mass that is below the starvation threshold and are thus infeasible. Arrows point to the predicted optimal mass from our model *M*_opt_ = 1.748 × 10^7^, which may serve as an evolutionary attractor for body mass. The black wedge points to the largest body mass known for terrestrial mammals (*Deinotherium* spp.) at 1.74 × 10^7^ g^31^

To assess the susceptibility of the resident species to competitive exclusion, we determine which consumer pushes the steady-state resource density R^*^ to lower values for a given value of *χ*, with the expectation that a population capable of surviving on lower-resource densities has a competitive advantage^58^. We find that for *M* ≤ 1.748 × 10^7^ g, having additional body fat (*χ* > 0) results in a lower steady-state resource density (*R*′^*^ < R^*^), such that the competitor has an intrinsic advantage over the resident species (Fig. 5). However, for *M* > 1.748 × 10^7^ g, leaner individuals (*χ* < 0) have lower-resource steady-state densities.

The observed switch in susceptibility as a function of *χ* at *M*_opt_ = 1.748 × 10^7^ g thus serves as an attractor, such that the NSM predicts organismal mass to increase if *M* < *M*_opt_ and decrease if *M* > *M*_opt_. This value is close to but smaller than the asymptotic upper bound for terrestrial mammal body size predicted by the NSM, and is remarkably close to independent estimates of the largest land mammals, the early Oligocene *Indricotherium* at ≈1.5 × 10^7^ g and the late Miocene *Deinotherium* at ≈1.74 × 10^7^ g^31^. Additionally, our calculation of *M*_opt_ as a function of mass-dependent physiological rates is similar to theoretical estimates of maximum body size^30^, and provides independent theoretical support for the observation of a “maximum body size attractor” explored by Alroy^29^.

An optimal size for mammals at intermediate body mass was predicted by Brown et al.^37^ based on reproductive maximization and the transition between hungry and full individuals. By coupling the NSM to resource dynamics as well as introducing an explicit treatment of storage, we show that species with larger body masses have an inherent competitive advantage for size classes up to *M*_opt_ = 1.748 × 10^7^ based on resource competition. Moreover, the mass distributions in ref. ^37^ show that intermediate mammal sizes have the greatest species diversity, in contrast to our efforts, which consider total biomass and predict a much larger *M*_opt_. Compellingly, recent work shows that many communities can be dominated by the biomass of the large^61^. While the state of the environment as well as the competitive landscape will determine whether specific body sizes are selected for or against^32^, we propose that the dynamics of starvation and recovery described in the NSM provide a general selective mechanism for the evolution of larger body size among terrestrial mammals.

## Discussion

The energetics associated with somatic maintenance, growth, and reproduction are important elements that influence the dynamics of all populations^10^. The NSM incorporates the dynamics of starvation and recovery that are expected to occur in resource-limited environments. We found that incorporating allometrically determined rates into the NSM predicts that (i) extinction risk is minimized, (ii) the derived steady states quantitatively reproduce Damuth’s law, and (iii) the selective mechanism for the evolution of larger body sizes agrees with Cope’s rule. The NSM offers a means by which the dynamic consequences of energetic constraints can be assessed using macroscale interactions between and among species.

## Methods

### Mechanisms of starvation and recovery

To understand the dynamics of starvation, recovery, reproduction, and resource competition, our framework partitions consumers into two classes: (a) a full class that is able to reproduce and, (b) a hungry class that experiences mortality at a given rate and is unable to reproduce. For the dynamics of growth, reproduction, and resource consumption, past efforts have combined the overall metabolic rate, as dictated by body size, with a growth rate that is dependent on resource abundance and, in turn, dictates resource consumption (see refs. ^3,62^ for a brief review of this perspective). This approach has been used to understand a range of phenomena including a derivation of ontogenetic growth curves from a partitioning of metabolism into maintenance and biosynthesis^3,63–65^ and predictions for the steady-state resource abundance in communities of cells^62^. Here, we leverage these mechanisms, combined with several additional concepts, to define our NSM.

We consider the following generalized set of explicit dynamics for starvation, recovery, reproduction, and resource growth and consumption

2 $$\begin{array}{*{20}{l}} {\dot F_{\mathrm{d}}} \hfill & = {\lambda _{\mathrm{max}}F_{\mathrm{d}} + \rho _{\mathrm{max}}R_{\mathrm{d}}H_{\mathrm{d}}/k - \sigma \left( {1 - \frac{{R_{\mathrm{d}}}}{C}} \right)F_{\mathrm{d}},} \hfill \\ {\dot H_{\mathrm{d}}} \hfill & { = \sigma \left( {1 - \frac{{R_{\mathrm{d}}}}{C}} \right)F_{\mathrm{d}} - \rho _{\mathrm{max}}R_{\mathrm{d}}H_{\mathrm{d}}/k - \mu H_{\mathrm{d}},} \hfill \\ {\dot R_{\mathrm{d}}} \hfill & { = \alpha R_{\mathrm{d}}\left( {1 - \frac{{R_{\mathrm{d}}}}{C}} \right) - } \hfill \\ {} \hfill & {\left[ {\left( {\frac{{\rho _{\mathrm{max}}R_{\mathrm{d}}}}{{Y_{\mathrm{H}}k}} + P_{\mathrm{H}}} \right)H_{\mathrm{d}} + \left( {\frac{{\lambda _{\mathrm{max}}}}{{Y_{\mathrm{F}}}} + P_{\mathrm{F}}} \right)F_{\mathrm{d}}} \right].} \hfill \end{array}$$

where each term has a mechanistic meaning that we detail below (we will denote the dimensional equations with the subscript _d_ before introducing the nondimensional form that is presented in the main text). In the above equations, *Y* represents the yield coefficient^66,67^ that is the quantity of resources required to build a unit of organism (gram of mammal produced per gram of resource consumed) and *P* is the specific maintenance rate of resource consumption (g resource · s^−1^ · g organism^−1^). If we pick *F*_d_ and *H*_d_ to have units of (g organisms ·  m^−2^), then all of the terms of $\dot R_{\mathrm{d}}$, such as $\frac{{\rho \left( {R_{\mathrm{d}}} \right)}}{Y}H_{\mathrm{d}}$, have units of (g resource ·  m^−2^ · s^−1^), the typical units of net primary productivity (NPP), a natural choice for $\dot R_{\mathrm{d}}$. This choice also gives *R*_d_ as (g m^−2^) that is also a natural unit and is simply the biomass density. In these units, *α *(s^−1^) is the specific growth rate of *R*_d_, *C* is the carrying capacity, or maximum density, of *R*_d_ in a particular environment, and *k* is the half-saturation constant (half the density of resources that would lead to maximum growth).

We can formally nondimensionalize this system by the rescaling of *F* = *fF*_d_, *H* = *fH*_d_, *R* = *qR*_d_, and *t* = *st*_d_, in which case our system of equations becomes

3 $$\begin{array}{l}\dot F = \frac{1}{s}\left[ {\lambda _{\mathrm{max}}F + \rho _{\mathrm{max}}\frac{R}{{qk}}H - \sigma \left( {1 - \frac{R}{{qC}}} \right)F} \right], \hfill \\ \dot H = \frac{1}{s}\left[ {\sigma \left( {1 - \frac{R}{{qC}}} \right)F - \rho _{\mathrm{max}}\frac{R}{{qk}}H - \mu H} \right], \hfill \\ \dot R = \frac{1}{s}\left[ {\alpha R\left( {1 - \frac{R}{{qC}}} \right) - \frac{q}{f}\left[ {\left( {\frac{{\rho _{\mathrm{max}}R}}{{Y_{\mathrm{H}}kq}} + P_{\mathrm{H}}} \right)H + \left( {\frac{{\lambda _{\mathrm{max}}}}{{Y_{\mathrm{F}}}} + P_{\mathrm{F}}} \right)F} \right]} \right].\end{array}$$

If we make the natural choice of *s* = 1, *q* = 1/*C*, and *f* = 1/*Y*_H_*k*, then we are left with

4 $$\begin{array}{*{20}{l}} {\dot F} \hfill & { = \lambda F + \xi \rho RH - \sigma \left( {1 - R} \right)F,} \hfill \\ {\dot H} \hfill & { = \sigma \left( {1 - R} \right)F - \xi \rho RH - \mu H,} \hfill \\ {\dot R} \hfill & { = \alpha R\left( {1 - R} \right) - \left( {\rho R + \delta } \right)H - \beta F} \hfill \end{array}$$

where we have dropped the subscripts on *λ*_max_ and *ρ*_max_ for simplicity, and *ξ* ≡ *C*/*k*, *δ* ≡ *Y*_H_*kP*_H_/*C*, and $\beta \equiv Y_{\mathrm{H}}k\left( {\frac{{\lambda _{\mathrm{max}}}}{{Y_{\mathrm{F}}}} + P_{\mathrm{F}}} \right)/C$. The above equations represent the system of equations presented in the main text.

### Analytical solution to the NSM

Equation (1) has three fixed points: two trivial fixed points at (F^*^, H^*^, R^*^) = (0, 0, 0) and (0, 0, 1), and one nontrivial, internal fixed point at

5 $$\begin{array}{*{20}{l}} {F}{^\ast } \hfill & { = \left( {\sigma - \lambda} \right)\frac{{\alpha \lambda \mu ^2(\mu + \xi \rho )}}{{A\left( {\lambda \rho B + \mu \sigma \left( {\beta \mu + \lambda \left( {\delta + \rho } \right)} \right)} \right)}},} \hfill \\ {H}{^\ast } \hfill & { = \left( {\sigma - \lambda } \right)\frac{{\alpha {\lambda}^{2}\mu (\mu + \xi \rho )}}{{A\left( {\lambda \rho B + \mu \sigma \left( {\beta \mu + \lambda \left( {\delta + \rho } \right)} \right)} \right)}},} \hfill \\ {R}{^\ast } \hfill & { = \left( {\sigma - \lambda } \right)\frac{\mu }{A}.} \hfill \end{array}$$

where *A* = (*λξρ* + *μσ*) and *B* = (*βμξ* + *δλξ *− *λμ*). The stability of this fixed point is determined by the Jacobian matrix **J**, with $J_{{{i}}j} = \partial \dot X_i/\partial X_j$, when evaluated at the internal fixed point, and **X** is the vector (*F*, *H*, *R*). The parameters in Eq. 1 are such that the real part of the largest eigenvalue of **J** is negative, so that the system is stable with respect to small perturbations from the fixed point. Because this fixed point is unique, it is the global attractor for all population trajectories for any initial condition where both the resource and consumer densities are nonzero.

### Metabolic scaling relationships

The scaling relation between an organism’s metabolic rate *B* and its body mass *M* at reproductive maturity is known to scale as *B* = *B*_0_*M*^*η*^, where the scaling exponent *η* is typically close to 2/3 or 3/4 for metazoans^47,56^, and has taxonomic shifts for unicellular species between *η* ≈ 1 in eukaryotes and *η* ≈ 1.76 in bacteria^3,68^.

Several efforts have shown how a partitioning of *B* between growth and maintenance purposes can be used to derive a general equation for both the growth trajectories and growth rates of organisms ranging from bacteria to metazoans^3,63–65,69,70^. This relation is derived from the simple balance condition $B_{\mathrm{0}}m^\eta = E_{\mathrm{m}}\dot m + B_{\mathrm{m}}m{\kern 1pt} ,$^3,63–65,69,70^ where *E*_m_ is the energy needed to synthesize a unit of mass, *B*_m_ is the metabolic rate to support an existing unit of mass, and *m* is the mass of the organism at any point in its development. This balance has the general solution^3,64,71^

6 $$\left( {\frac{{m\left( t \right)}}{M}} \right)^{1 - \eta } = 1 - \left[ {1 - \left( {\frac{{m_{\mathrm{0}}}}{M}} \right)^{1 - \eta }} \right]\mathrm{e}^{ - a\left( {1 - \eta } \right)t/M^{1 - \eta }},$$

where, for *η* < 1, *M* = (*B*_0_/*B*_m_)^1/(1−*η*)^ is the asymptotic mass, *a* = *B*_0_/*E*_m_, and *m*_0_ is mass at birth itself varying allometrically. We now use this solution to define the timescale for reproduction and recovery from starvation (Fig. 2; see ref. ^64^ for a detailed presentation of these timescales). The time that an organism takes to reach a particular mass *εM* is given by the timescale

7 $$\tau \left( \varepsilon \right) = {\rm{ln}}\left[ {\frac{{1 - \left( {m_{\mathrm{0}}/M} \right)^{1 - \eta }}}{{1 - \varepsilon ^{1 - \eta }}}} \right]\frac{{M^{1 - \eta }}}{{a\left( {1 - \eta } \right)}},$$

where we define values of *ε* below to describe a variety of timescales, along with the rates related to *τ*. For example, the rate of reproduction is given by the timescale to go from the birth mass to the adult mass. The time to reproduce is given by Eq. 7 as *t*_*λ*_ = *τ*(*ε*_*λ*_), where *ε*_*λ*_ is the fraction of the asymptotic mass where an organism is reproductively mature and should be close to one (typically *ε*_*λ*_ ≈ 0.95^63^). Our reproductive rate, *λ*, is a specific rate, or the number of offspring produced per time per individual, defined as $\dot F = \lambda F$. In isolation, this functional form gives the population growth $F\left( t \right) = F_{\mathrm{0}}e^{\lambda t}$ that can be related to the reproductive timescale by assuming that when *t* = *t*_*λ*_, it is also the case that *F* = *νF*_0_, where *ν* is the number of offspring produced per reproductive cycle. Following this relationship, the growth rate is given by *λ* = ln(*ν*)/*t*_*λ*_, which is the standard relationship^70^ and will scale as *λ* ∝ *M*^*η*−1^ for *M* >> *m*_0_ and any constant value of *ε*_*λ*_^3,63–65,69^.

The rate of recovery *ρ* = 1/*t*_*ρ*_ requires that an organism accrues sufficient tissue to transition from the hungry to the full state. Since only certain tissues can be digested for energy (e.g., the brain should not be degraded to fuel metabolism), we define the rates for starvation, death, and recovery by the timescales required to reach, or return from, specific fractions of the replete-state mass (Table 1). We define *m*_*σ*_ = *ε*_*σ*_*M*, where *ε*_*σ*_ < 1 is the fraction of replete-state mass where reproduction ceases. This fraction will deviate from a constant if tissue composition systematically scales with adult mass. For example, making use of the observation that body fat in mammals scales with overall body size according to *M*_fat_ = *f*_0_*M*^*γ*^ and assuming that once this mass is fully digested and the organism starves, this would imply that *ε*_*σ*_ = 1 − *f*_0_*M*^*γ*^/*M*. It follows that the recovery timescale, *t*_*ρ*_, is the time to go from mass *m* = *ε*_*σ*_*ε*_*λ*_*M* to *m* = *ε*_*λ*_*M* (Fig. 2). Using Eqs. 6 and 7, this timescale is given by simply considering the growth curve starting from a mass of $m_{\mathrm{0}}' = \varepsilon _{\mathrm{\sigma }}\varepsilon _{\mathrm{\lambda }}M$, in which case

8 $$t_{\mathrm{\rho }} = {\rm{ln}}\left[ {\frac{{1 - \left( {\varepsilon _{\mathrm{\sigma }}\varepsilon _{\mathrm{\lambda }}} \right)^{1 - \eta }}}{{1 - \varepsilon _\lambda ^{1 - \eta }}}} \right]\frac{{M^{1 - \eta }}}{{a'\left( {1 - \eta } \right)}}$$

where $a' = B_{\mathrm{0}}/E_{\mathrm{m}}'$ accounts for possible deviations in the biosynthetic energetics during recovery. It should be noted that more complicated ontogenetic models explicitly handle storage, whereas this feature is implicitly covered by the body fat scaling in our framework.

To determine the starvation rate, *σ*, we are interested in the time required for an organism to go from a mature adult that reproduces at rate *λ*, to a reduced-mass hungry state where reproduction is impossible. For starving individuals, we assume that an organism must meet its maintenance requirements by using the digestion of existing mass as the sole energy source. This assumption implies the metabolic balance $\dot mE_{\mathrm{m}}' = - B_{\mathrm{m}}m$ or $\dot m = - a'm/M^{1 - \eta }$, where $E_{\mathrm{m}}'$ is the amount of energy stored in a unit of existing body mass, which differs from *E*_m_, the energy required to synthesize a unit of biomass. Given the replete mass, *M*, of an organism, the above energy balance prescribes the mass trajectory of a nonconsuming organism: $m\left( t \right) = Me^{ - a't/M^{1 - \eta }}$. The timescale for starvation is given by the time it takes *m*(*t*) to reach *ε*_*σ*_*M*, which gives

9 $$t_{\mathrm{\sigma }} = - \frac{{M^{1 - \eta }}}{{a'}}{\rm{ln}}\left( {\varepsilon _{\mathrm{\sigma }}} \right).$$

The starvation rate is then *σ* = 1/*t*_*σ*_, which scales with replete-state mass as $-M^{\eta - 1}{\rm{ln}}\left( {1 - f_{\mathrm{0}}M^\gamma /M} \right)^{-1}$. An important feature is that *σ* does not have a simple scaling dependence on *λ*, which is important for the dynamics that we later discuss.

The time to death should follow a similar relation, but defined by a lower fraction of replete-state mass, *m*_*μ*_ = *ε*_*μ*_*M* where *ε*_*μ*_ < *ε*_*σ*_. Suppose, for example, that an organism dies once it has digested all fat and muscle tissues, and that muscle tissue scales with body mass according to *M*_musc_ = *u*_0_*M*^*ζ*^. This gives $\varepsilon _{\mathrm{\mu }} = 1 - \left( {f_{\mathrm{0}}M^\gamma + u_{\mathrm{0}}M^\zeta } \right)/M$. Muscle mass has been shown to be roughly proportional to body mass^72^ in mammals and thus *ε*_*μ*_ is merely *ε*_*σ*_ minus a constant. The time to go from starvation to death is the total time to reach *ε*_*μ*_*M* minus the time to starve, or $t_{\mathrm{\mu }} = - M^{1 - \eta }{\rm{ln}}\left( {\varepsilon _{\mathrm{\mu }}} \right)/a' - t_{\mathrm{\sigma }}$, and *μ* = 1/*t*_*μ*_.

### Parameter values and estimates

All of the parameter values employed in our model have either been directly measured in previous studies or can be estimated from combining several previous studies. Below, we outline previous measurements and simple estimates of the parameters.

Metabolic rate has been generally reported to follow an exponent close to *η* = 0.75^63–65^. We make this assumption in the current paper, although alternate exponents, which are known to vary between roughly 0.25 and 1.5 for single species^64^, could be easily incorporated into our framework. The exponent not only defines several scalings in our framework, but also the value of the metabolic normalization constant, *B*_0_, given a set of data. For mammals, the metabolic normalization constant has been reported to vary between 0.018 W g^−0.75^ and 0.047 (W g^−0.75^; refs. ^63,65^, where the former value represents basal metabolic rate and the latter represents the field metabolic rate). We employ the field metabolic rate for our NSM model that is appropriate for active mammals (Table 1).

An important feature of our framework is the starting size, *m*_0_, of a mammal that adjusts the overall timescales for reproduction. This starting size is known to follow an allometric relationship with adult mass of the form *m*_0_ = *n*_0_*M*^*υ*^ where estimates for the exponent range between 0.71 and 0.94 (see ref. ^73^ for a review). We use *m*_0_ = 0.097*M*^0.92^ ^74^ that encompasses the widest range of body sizes^73^.

The energy to synthesize a unit of biomass, *E*_m_, has been reported to vary between 1800 and 9500 (J g^−1^)^63–65^ in mammals with a mean value across many taxonomic groups of 5774 (J g^−1^)^64^. The unit energy available during starvation, *E*′, could range between 7000 (J g^−1^), the return of the total energy stored during ontogeny to a biochemical upper bound of *E*′ = 36,000 (J g^−1^) for the energetics of palmitate^65,75^. For our calculations, we use the measured value for bulk tissues of 7000 which assumes that the energy stored during ontogeny is returned during starvation^65^.

For the scaling of body composition, it has been shown that fat mass follows *M*_fat_ = *f*_0_*M*^*γ*^, with measured relationships following 0.018*M*^1.25^ ^76^, 0.02*M*^1.19^ ^77^, and 0.026*M*^1.14^ ^78^. We use the values from^77^ that falls in the middle of this range. Similarly, the muscle mass follows *M*_musc_ = *u*_0_*M*^*ζ*^ with *u*_0_ = 0.383 and *ζ* = 1.00^78^.

Typically, the value of *ξ* = *C*/*k* should roughly be 2. The values of *ρ*, *λ*, *σ*, and *μ* are all simple rates (note that we have not rescaled time in our nondimensionalization) as defined in the main text. Given that our model considers transitions over entire stages of ontogeny or nutritional states, the value of *Y* must represent yields integrated over entire life stages. Given an energy density of *E*_d_ = 18,200 (J g^−1^) for grass,^79^ the maintenance value is given by *P*_F_ = *B*_0_*M*^3/4^/*ME*_d_, and the yield for a full organism will be given by *Y*_F_ = *ME*_d_/*B*_*λ*_ (g individual g grass ^−1^), where *B*_*λ*_ is the lifetime energy use for reaching maturity given by

10 $$B_{\mathrm{\lambda }} = \mathop {\int}\limits_{\mathrm{0}}^{t_{\mathrm{\lambda }}} {B_{\mathrm{0}}m\left( t \right)^\eta {\rm{d}}t.}$$

Similarly, the maintenance resource consumption rate for hungry individuals is *P*_H_ = *B*_0_(*ε*_*σ*_*M*)^3/4^/(*ε*_*σ*_*M*)*E*_d_, and the yield for hungry individuals (representing the cost on resources to return to the full state) is given by *Y*_H_ = *ME*_d_/*B*_*ρ*_ where

11 $$B_{\mathrm{\rho }} = \mathop {\int}\limits_{{\mathrm{\tau }}\left( {\varepsilon _{\mathrm{\sigma }}\varepsilon _{\mathrm{\lambda }}} \right)}^{t_{\mathrm{\lambda }}} {B_{\mathrm{0}}m\left( t \right)^\eta {\rm{d}}t.}$$

Taken together, these relationships allow us to calculate *ρ*, *δ*, and *β*.

Finally, the value of *α* can be roughly estimated by the NPP divided by the corresponding biomass densities. From the data in Ref. ^80^, we estimate the value of *α* to range between 2.81 × 10^−10^ (s^−1^) and 2.19 × 10^−8^ (s^−1^) globally. It should be noted that the value of *α* sets the overall scale of the F^*^ and H^*^ steady states along with *B*_tot_ for each type. As such, we use *α* as our fit parameter to match these steady states with the data from Damuth^25^. We find that the best fit is *α* = 9.45 × 10^−9^ (s^−1^) that compares well with the calculated range above. However, two points are important to note here: first, our framework predicts the overall scaling of F^*^ and H^*^ independently of *α* and this correctly matches data, and second, both the asymptotic behavior and slope of F^*^ and H^*^ are independent of *α*, such that our prediction of the maximum mammal size does not depend on *α*.

### Rate equations for competitors with modified body mass

A resident population with mass *M* competes for resources with a closely related species with an altered mass *M*′ = *M*(1 + *χ*) where *χ* varies between *χ*_min_ < 0 and *χ*_max_ > 0, where *χ* < 0 denotes a leaner competitor and *χ* > 0 denotes a competitor with additional reserves of body fat. Importantly, we assume that the competing and resident individuals have the same quantity of nonfat tissues. For the allowable values of *χ*, the adjusted mass should exceed the amount of body fat, 1 + *χ* > *ε*_*σ*_, and the adjusted time to reproduce must be positive, which given our solution for *τ*(*ε*) (Eq. 7), implies that $1 - \varepsilon _{\mathrm{\lambda }}^{1 - \eta }\left( {1 + \chi } \right)^{1 - \eta } > 0$. Together, these conditions imply that *χ *∈ (−*f*_0_*M*^*γ*−1^,1/*ε*_*λ *_− 1) where the upper bound approximately equals 0.05.

Although the starved state of competing organisms remains unchanged, the rate of starvation from the modified full state to the starved state, the rate of recovery from the starved state to the modified full state, and the maintenance rates of both will be different, such that *σ*′ = *σ*(*M*′), *ρ*′ = *ρ*(*M*′), *β*′ = *β*(*M*′), and *δ*′ = *δ*(*M*′). Rates of starvation and recovery for the competing population are derived by adjusting the starting or ending state before and after starvation and recovery, leading to the following timescales:

12 $$\begin{array}{l}t_{{{\mathrm \sigma}'}} = - \frac{{M^{1 - \eta }}}{{a'}}{\rm{ln}}\left( {\frac{{\varepsilon _\sigma }}{{\chi + 1}}} \right),\\ t_{{{\mathrm \rho}'}} = {\rm{ln}}\left( {\frac{{1 - \left( {\varepsilon _\lambda \varepsilon _\sigma } \right)^{1/4}}}{{1 - \left( {\varepsilon _\lambda \left( {\chi + 1} \right)} \right)^{1/4}}}} \right)\frac{{M^{1 - \eta }}}{{a'\left( {1 - \eta } \right)}}.\end{array}$$

The maintenance rates for the competing population require more careful consideration. First, we must recalculate the yield *Y*, as it must now be integrated over life stages that have also been slightly modified by the addition or subtraction of body fat reserves. Given an energy density of *E*_d_ = 18,200 (J g^−1^) for grass^80^, the maintenance value of the invading population is given by *P*_F_ = *B*_0_(1 + *χ*)*M*^3/4^/(1 + *χ*)*ME*_d_, and the yield for a full organism will be given by $Y_{\mathrm{F}} = (1 + \chi )ME_{\mathrm{d}}/B_{\mathrm{\lambda }}'$ (g individual g grass ^−1^) where $B_{\mathrm{\lambda }}'$ is the lifetime energy use for the competing population reaching maturity given by

13 $${B'}_{\mathrm{\lambda }} = \mathop {\int}\limits_{\mathrm{0}}^{t_{{\lambda}'}} {B_{\mathrm{0}}m\left( t \right)^\eta {\rm{d}}t.}$$

where

14 $$t_{{{\mathrm \lambda'}}} = \frac{{M^{1 - \eta }}}{{a\left( {1 - \eta } \right)}}{\rm{ln}}\left( {\frac{{1 - \left( {m_0/M} \right)^{1 - \eta }}}{{1 - \left( {\varepsilon _\lambda \left( {1 + \chi } \right)} \right)^{1 - \eta }}}} \right).$$

Note that we do not use this timescale to determine the reproductive rate of the competitor—which is assumed to remain the same as the resident population—but only to calculate the lifetime energy use. Similarly, the maintenance for hungry individuals $P_{\mathrm{H}}' = B_{\mathrm{0}}(\varepsilon _{\mathrm{\sigma }}(1 + \chi )M)^{3/4}/(\varepsilon _{\mathrm{\sigma }}(1 + \chi )M)E_{\mathrm{d}}$ and the yield for hungry individuals (representing the cost on resources to return to the full state) is given by $Y_{{\mathrm H}'} = (1 + \chi )ME_{\mathrm{d}}/B_{{\mathrm \rho }'}$ where

15 $${{B}'}_{\mathrm{\rho }} = \mathop {\int}\limits_{{\mathrm{\tau }}\left( {\varepsilon _{\mathrm{\sigma }}\varepsilon _{\mathrm{\lambda }}} \right)}^{t_{{{\mathrm \lambda}'}}} {B_{\mathrm{0}}m\left( t \right)^\eta {\rm{d}}t.}$$

Finally, we can calculate the maintenance of the competitors as

16 $$\begin{array}{l}\delta' = P'_{\mathrm{H}}Y'_{\mathrm{H}}/\xi \\ \beta' = \left( {\frac{{\lambda _{\mathrm{max}}}}{{Y'_{\mathrm{F}}}} + P'_{\mathrm{F}}} \right)Y'_{\mathrm{H}}/\xi .\end{array}$$

To determine whether or not the competitor or resident population has an advantage, we compute R*(*M*) and R*(*M*′ = *M*(1 + *χ*)) for values of *χ *∈ (−*f*_0_*M*^*γ*−1^,1/*ε*_*λ *_− 1), and the competing population is assumed to have an advantage over the resident population if R*(*M*′) < R*(*M*).

### Data availability

Previously published data from Damuth (available in ref. ^25^) were used for comparison with the model, and published parameter values are given in Table 1.

## Electronic supplementary material

Supplementary Information
