# Supplementary material for: Dynamics of starvation and recovery predict extinction risk and both Damuth’s law and Cope’s rule
Source: Nat Commun. 2018 Feb 13;9:657. doi: 10.1038/s41467-018-02822-y (PMC5811595; doi:10.1038/s41467-018-02822-y)
Supplement: Supplementary file 1 — Supplementary Information [file 41467_2018_2822_MOESM1_ESM.pdf]

**Supplementary Note 1: Sensitivity to additional death terms**

It should be noted that our set of dynamics (Eqs. 2 and 4, main text) could include a constant death term of the form $-d_F F$ and $-d_H H$ to represent death not directly linked to starvation. Adding terms of this form to our model would simply adjust the effective value of $\lambda$ and $\mu$, and we could rewrite Eq. 4 with $\lambda' = \lambda - d$ and $\mu' = \mu - d$. These substitutions would not alter the functional form of our model nor the steady-states and qualitative results, however the quantitative values could shift based on the size of $d$ relative to $\lambda$ and $\mu$.

Survivorship has a well-known functional form which changes systematically with size (e.g.[17]). Typically survivorship is defined using the Gompertz curve

$$F = F_0 e^{(c_0/c_1)\left(1-e^{c_1 t}\right)} \tag{1}$$

where the parameters have the following allometric dependencies on adult mass $c_0 = a_0 M^{b_0}$ and $c_1 = a_1 M^{b_1}$, with $a_0 = 1.88 \times 10^{-8}$ (s g$^{-b_0}$), $b_0 = -0.56$, $a_1 = 1.45 \times 10^{-7}$ (s g$^{-b_1}$), and $b_1 = -0.27$ (see[17] for a review).

We are interested in the specific death rate of the form $\dot{F} = -dF$, and using the derivative of Eq. 1 we find that $d = c_0 e^{c_1 t}$. Our model considers the average rates over a population and lifecycle and the average death rate is given by

$$\bar{d} = \frac{1}{t_{\text{exp}}} \int_0^{t_{\text{exp}}} c_0 e^{c_1 t} dt \tag{2}$$

$$= \frac{c_0\left(e^{c_1 t_{\text{exp}}} - 1\right)}{c_1 t_{\text{exp}}} \tag{3}$$

where $t_{\text{exp}}$ is the expected lifespan following the allometry of $t_{\text{exp}} = a_2 M^{b_2}$ with $a_2 = 4.04 \times 10^6$ (s g$^{-b_2}$) and $b_2 = 0.30$ [17,18]. Given the allometries above we have that

$$\bar{d} = \frac{a_0\left(e^{a_1 a_2 M^{b_1+b_2}} - 1\right) M^{b_0-b_1-b_2}}{a_1 a_2} \tag{4}$$

which scales roughly like $M^{b_0}$ because $b_1$ and $b_2$ are close in value but opposite in sign. In Supplementary Figure 1 we compare the value of $\bar{d}$ to the reproductive, $\lambda$, and starvation-based mortality, $\mu$, rates. The values of $\bar{d}$ are orders of magnitude smaller than these rates for all mammalian masses, and thus, adding this non-starvation based death rate to our model does not shift our results within numerical confidence.

**Supplementary Note 2: NSM and the energy equivalence hypothesis**

The energy equivalence hypothesis is based on the observation that if one assumes that the total metabolism of an ecosystem $B_{\text{tot}}$ is equally partitioned between all species ($B_i$, the total metabolism of one species, is a constant), then the abundances should follow $N(M)\,B(M) = B_i$ implying that $N(M) \propto M^{-\eta}$, where $\eta$ is the metabolic scaling exponent[19,20]. As $\eta \approx 3/4$ this hypothesis is consistent with Damuth's law[19]. However, the actual equivalence of energy usage of diverse species has not been measured at the population level for a variety of whole populations. Supplementary Figure 2 recasts the results of the NSM in terms of this hypothesis and shows that $F^* B$ is nearly constant over the same range of mammalian sizes up to the asymptotic behavior for the largest terrestrial mammals.

**Supplementary Note 3: Application of NSM limits to aquatic mammals** A theoretical upper bound on mammalian body size is given by $\epsilon_\sigma = 0$, where mammals are entirely composed of metabolic reserves, and this occurs at $M = 8.3 \times 10^8$ (g), or 120 times the mass of a male African elephant. We note this particular limit as it may have future relevance to considerations of the ultimate constraints on aquatic mammals.

## Supplementary Figures

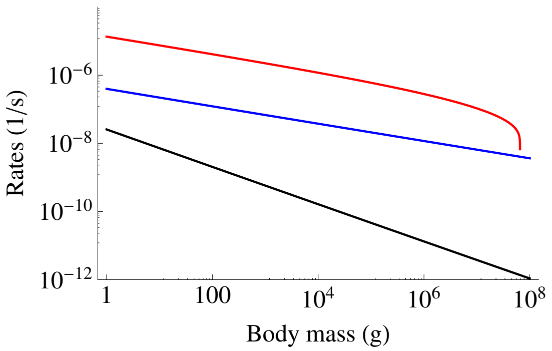


Supplementary Figure 1: The rates of reproduction $\lambda$ (blue), starvation-based mortality $\mu$ (red), and survivorship-based death $\bar{d}$ (black) as a function of adult mass.

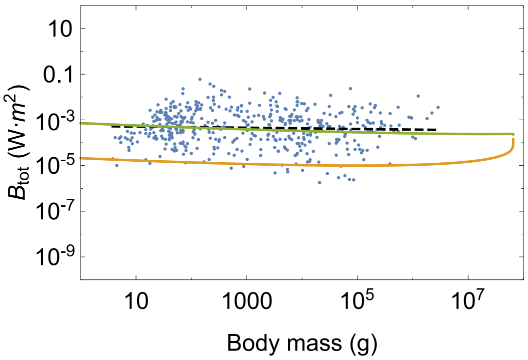


Supplementary Figure 2: Total energetic use $B_{\text{tot}}$ of consumer populations at the steady state as a function of body mass ($F^*$ is shown in green and $H^*$ in orange). The data are from Damuth[21] and have been converted to total population metabolism using the allometric relationships for metabolic rate (e.g. Refs.[3–5]).

## Supplementary References

---


[1] Kempes, C. P., Dutkiewicz, S. & Follows, M. J. Growth, metabolic partitioning, and the size of microorganisms. *PNAS* **109**, 495–500 (2012).

[2] Kempes, C. P., Okegbe, C., Mears-Clarke, Z., Follows, M. J. & Dietrich, L. E. Morphological optimization for access to dual oxidants in biofilms. *Proceedings of the National Academy of Sciences* **111**, 208–213 (2014).

[3] West, G. B., Brown, J. H. & Enquist, B. J. A general model for ontogenetic growth. *Nature* **413**, 628–631 (2001).

[4] Moses, M. E. *et al.* Revisiting a model of ontogenetic growth: Estimating model parameters from theory and data. *American Naturalist* **171**, 632–645 (2008).

[5] Hou, C. *et al.* Energy uptake and allocation during ontogeny. *Science* **322**, 736–739 (2008).

[6] Pirt, S. The maintenance energy of bacteria in growing cultures. *Proceedings of the Royal Society of London B: Biological Sciences* **163**, 224–231 (1965).

[7] Heijnen, J. & Roels, J. A macroscopic model describing yield and maintenance relationships in aerobic fermentation processes. *Biotechnology and Bioengineering* **23**, 739–763 (1981).

[8] Peters, R. H. *The Ecological Implications of Body Size*, vol. 2 (Cambridge University Press, Cambridge, 1986).

[9] Blueweiss, L. *et al.* Relationships between body size and some life history parameters. *Oecologia* **37**, 257–272 (1978).

[10] Stryer, L. *Biochemistry, Fourth Edition* (W.H. Freeman and Company, New York, 1995).

[11] Dunbrack, R. L. & Ramsay, M. A. The allometry of mammalian adaptations to seasonal environments: A critique of the fasting endurance hypothesis. *Oikos* **66**, 336–342 (1993).

[12] Lindstedt, S. L. & Boyce, M. S. Seasonality, fasting endurance, and body size in mammals. *Am. Nat.* **125**, 873–878 (1985).

[13] Lindstedt, S. L. & Schaeffer, P. J. Use of allometry in predicting anatomical and physiological parameters of mammals. *Lab. Anim.* **36**, 1–19 (2002).

[14] Estermann, B. L., Wettstein, H.-R., Sutter, F. & Kreuzer, M. Nutrient and energy conversion of grass-fed dairy and suckler beef cattle kept indoors and on high altitude pasture. *Animal Research* **50**, 477–493 (2001).

[15] Michaletz, S. T., Cheng, D., Kerkhoff, A. J. & Enquist, B. J. Convergence of terrestrial plant production across global climate gradients. *Nature* **512**, 39–43 (2014).

[16] Damuth, J. Interspecific allometry of population density in mammals and other animals: the independence of body mass and population energy-use. *Biological Journal of the Linnean Society* **31**, 193–246 (1987).

[17] Calder, W. A. *Size, function, and life history* (Harvard University Press, 1984).

[18] Damuth, J. Analysis of the preservation of community structure in assemblages of fossil mammals. *Paleobiology* **8**, 434–446 (1982).

[19] Allen, A. P., Brown, J. H. & Gillooly, J. F. Global biodiversity, biochemical kinetics, and the energetic-equivalence rule. *Science* **297**, 1545–1548 (2002).

[20] Enquist, B. J., Brown, J. H. & West, G. B. Allometric scaling of plant energetics and population density. *Nature* **395**, 163–165 (1998).

[21] Damuth, J. Interspecific allometry of population density in mammals and other animals: the independence of body mass and population energy-use. *Biol. J. Linn. Soc.* **31**, 193–246 (1987).
